# Problem solving skills versus knowledge acquisition: the historical dispute that split problem-based learning into two camps

**DOI:** 10.1007/s10459-018-9835-0

**Published:** 2018-05-30

**Authors:** Virginie F. C. Servant-Miklos

**Affiliations:** 1 Aalborg University, Rendsburggade 14, 9000 Aalborg, Denmark; 2 Erasmus University College, Nieuwemarkt 1A, 3011 HP Rotterdam, The Netherlands

**Keywords:** Problem-based learning, History, Constructivism, Hypothetico-deduction

## Abstract

This paper sheds light on an intellectual dispute on the purpose of problem-based learning that took place in the 1970s between two major figures in the history of PBL: Howard S Barrows from McMaster University and Henk Schmidt from Maastricht University. Using historical evidence from archive materials, oral history accounts and contemporary publications, the paper shows that at the core of the dispute was their divergent understanding of cognitive psychology. On the one hand, Barrows espoused hypothetico-deduction, and on the other, Henk Schmidt was a proponent of constructivism. The paper shows how the dispute played out both in the scientific literature and in the divergent practice of PBL at McMaster and Maastricht and continues to affect the way PBL is done today.

## Introduction

In 2009, Schmidt et al. (2009) acknowledged that there was not one but several types of problem-based learning curricula being used by medical schools in North America and beyond. Primarily, they distinguished between what they called Type 1 curricula, in which students are asked to generate a “mental model” of phenomena underlying a problem, and a Type 2, in which students “play doctor”, focusing on problem-solving and clinical reasoning skills. For the sake of simplicity, we shall refer to the former as the Knowledge Acquisition model, and the latter as the Problem-Solving Skills model. What Schmidt and colleagues did not explain is how, issued from a single source, namely McMaster University Medical School’s 1969 pioneering programme, the world of PBL came to be divided along this fault-line. The beginnings of PBL were recently the subject of extensive research and shall not be covered in detail here (Servant 2016). The story can be summarised as follows: in the period after the second World War, higher education experienced an unparalleled growth around the western world, which, combined with bountiful financial resources and a rising tide of anti-authoritarianism, contributed to the birth and development of many innovative higher education programmes in various disciplines. In Germany and Denmark, problem-oriented education grounded in critical theory emerged in social sciences and humanities, and later engineering, as a challenge to mainstream didactics (Servant-Miklos and Spliid 2017); in business education, the Harvard Case Method gained international traction (Garvin 2003); in medical education, Western Reserve University pioneered an organ systems-based approach, a direct ancestor to PBL (Williams 1980). Between 1966 and 1972 a group of creative Canadian medical educators assembled around McMaster Medical School’s founding Dean Dr. John Evans with the mission to start a new undergraduate medical education programme. They took the medical education world by storm when instead of opening a traditional school, they decided to develop a small-group, self-directed, problem-based learning curriculum (Spaulding 1991). Their students began their learning with biomedical problems under the guidance of a tutor who acted as a process guide rather than a lecturer, leaving students to do most of the studying in their own time (Spaulding 1968). By the time of Schmidt’s article, over 500 medical schools were using some form of PBL (Moust et al. 2007), the majority using the Problem-Solving Skills version, and a substantial minority the Knowledge Acquisition version. This division is interesting considering the substantial support that the latter position has gained in the scientific literature, often at the expense of the former—a support that this paper will surely reinforce. How did these two iterations emerge from the McMaster experiment? What is the difference between these two versions, and how does this play out in terms of the way PBL is conducted? Why does this difference matter for medical education? Using historical data from oral history interviews, archives from McMaster University and Maastricht University and contemporary publications, this paper will try to answer these questions and shed light on a little known but highly significant divide in medical education.

The research for this paper was done using an inductive and hermeneutic approach to historical data in provenance from three types of primary sources that were triangulated to make sense of the historical events and the meanings ascribed to them by those who experienced them. These three sources were oral history accounts from primary witnesses, who were interviewed in English on site at McMaster and Maastricht Universities; archival records from McMaster University, Maastricht University, the Rijksarchief in Limburg and the private collections of former teachers, students and managers and both institutions, and contemporary publications and out-of-print books and journals that were acquired via the second-hand market or directly from the authors. Events, their meanings and interpretations were given weight according to how many independent sources could support the interpretation. Where a conflict emerged between the recollections of a witness in an oral history account and a written record, the written record was given precedence unless there was overwhelming oral historical evidence to the contrary. In writing this paper, the focus was on interpreting and analysing an important historical development rather than on providing a descriptive history of what happened at McMaster and Maastricht.

### Why did two iterations of PBL emerge from the original McMaster model?

To understand how two different interpretation of PBL emerged from the original experiment at McMaster, it is important to understand that the 1969 McMaster programme was not designed as a realisation of educational theory principles, as has often been claimed. The five founding fathers of PBL at McMaster University were pioneers and innovators, but not education theorists. In 1966, Dr. John Evans drafted a one-page bullet-pointed list of ideas which became the founding principles of PBL, but he never wrote anything significant to justify his choice of items for the list (Evans 1966). The list read as follows:

“The Following is an outline of the objectives for the McMaster M.D. Programme as expressed in terms of knowledge, abilities and attitudes that McMaster would like a graduate of the programme to have acquired or developed:

1. The ability to identify and define health problems, and search for information to resolve or manage these problems.
2. Given a health problem, to examine the underlying physical or behavioural mechanisms. […]
3. The ability to recognize, maintain and develop personal characteristics and attitudes required for professional life […]
4. The clinical skills and methods required to define and manage health problems of patients, including their physical, emotional and social aspects.
5. The ability to become a self-directed learner, recognizing personal education needs, selecting appropriate learning resources and evaluating progress.
6. To assess professional activity, both personal and that of other health professionals
7. To function as a productive member of a small group, which is engaged in learning, research or healthcare.
8. To be aware of and able to work in a variety of health care settings.”

As far as we know, his main source of inspiration was the Flexner report (McAuley 1979), but extracting from this anything more than general statements about the outdatedness of lecture-based medical education would be a stretch. Evans’ right-hand man Bill Spaulding occasionally mused about the 16th Century humanist Johannes Comenius (Spaulding 1968), but Spaulding’s role as the Chair of McMaster’s Education Committee was more that of a nuts-and-bolt planner than an education philosopher. Jim Anderson, possibly the most creative of the founding fathers, may have been inspired by humanistic principles, but he was really an inspired anatomist, not an education psychologist (Barrows 1996a, b). Neither of the final two members of the Education Committee—Fraser Mustard and Bill Walsh—had read much beyond what was widely circulating in higher education circles at the time; namely Mager’s *Behaviour Objectives* (Mager 1962) and the work of Knowles on self-directed learning (Knowles 1975). The lack of strong and coherent theoretical underpinnings for the programme meant that the McMaster experiment was more of a trial-and-error process in constant development than an application of cleverly crafted educational ideas. The fact that the term “problem-based learning” wasn’t coined in print until 1974 (Neufeld and Barrows 1974), and not by any of the founding fathers, goes to show just how uninterested the latter were in making grand jargonistic statements about what they were doing. A review of contemporary journal publications (Campbell 1973; Neufeld and Spaulding 1973; Spaulding and Neufeld 1973; Spaulding 1969) shows three things: firstly, that very little was published about PBL in its early years; secondly, that what was written tended to be by faculty who were not part of the original education committee; and thirdly, that the articles that were published tended to be descriptive rather than analytic. This meant that there was no definitive statement of what PBL was or what it was for, and when the founding fathers left the Education Committee—beginning with John Evans who resigned as Dean in 1972—the concept of PBL took on a life of its own under the pen of later prophets who filled the theoretical void with their own, often conflicting interpretations of what PBL was about.

The dispute at the heart of the division between the Problem-Solving Skills and the Knowledge Acquisition Approach is precisely the product of the theoretical chasm left by McMaster’s founders. This dispute played out in the late 1970s and 1980s between two of PBL’s most prolific theorists: Dr. Howard Barrows, a neurologist originally from California who came to McMaster on sabbatical in 1968 and joined the faculty roster from 1971 until 1981; and Henk Schmidt, a Dutch psychologist who was hired at the start of Maastricht University’s PBL programme in 1974 as part of the Department of Education Research and Development. The Maastricht programme was adapted from McMaster but sported some notable differences, such as the inclusion of systematic tutor and student training (Schmidt 1977a, b), the development of a “skills lab” for clinical skills training (Bartholomeus 1977), the codification of the PBL method into seven steps (Schmidt et al. 1979), the use of biomedical problems rather than (only) patient cases (Schmidt et al. 1979), and the allocation of research funds for a Department of Educational Research and Development (Rijksuniversiteit Limburg 1972). Although this department was officially run by the psychologist and assessment specialist Dr. Wynand Wijnen, in practice most of the early research on PBL was done by Henk Schmidt and his colleague Peter Bouhuijs. Howard Barrows was an occasional visitor and advisor to Maastricht, and Schmidt an occasional guest at McMaster, but their divergence of perspective on PBL played out mostly on paper. Both authors wrote their first major book on PBL in 1980 (Barrows and Tamblyn 1980; Schmidt and Bouhuijs 1980), and by that time their academic differences had already crystallised into an unbridgeable epistemological gulf. This means that Barrows and Schmidt’s understanding of what knowledge is, how it is constructed and how it is used in problem-solving was not only different, but fundamentally contradictory, such that the two positions could not be reconciled in the middle—one cannot take both positions at once, as the next section will explain.

### What is the difference between the problem-solving skills and the knowledge acquisition approach?

The crux of the intellectual dispute behind the two versions of PBL lies in two differing interpretations of what happens to the learner who is engaged in problem-based learning. On the one hand, some, led by Barrows, believed that the learners in PBL were honing “clinical reasoning skills” (Barrows and Tamblyn 1980) through a process called “hypothetico-deduction” (Elstein et al. 1978). We refer to this as the Problem-Solving Skills approach to PBL. Others, led by Schmidt, believed that learners in PBL were triggered by context-bound problems to understand the phenomena underlying the situation described in therein. We refer to this as the Knowledge Acquisition approach to PBL.

Both approaches are the product of the Cognitive Revolution in psychology, which began in 1956. The ‘50s were the heyday of behaviourism, but at an MIT symposium which brought together figures such as Jerome Bruner, Allen Newell, Herbert Simon and Noam Chomsky, a new interpretation of psychology was born that was based not on the study of behaviours and conditioning, but of mental processes instead (Miller 2003). While Bruner and Millers’ early work on cognition proceeded in relative isolation, the straw that broke the behaviourist camel’s back was Chomsky’s 1956 paper on linguistics (Chomsky 1956, 1967); it demolished the behaviourist understanding of language acquisition by showing that a purely behavioural account could not explain grammar acquisition. Chomsky’s pioneering paper paved the way for what some regard as one of the first works of cognitive psychology:* A Study of Thinking* by Bruner, Goodnow and Austin, from 1956 (Bechtel et al. 2001; Bruner et al. 1956).

And yet, the very people who broke the hold of behaviourism on American psychology in 1956 were also the authors of a schism that divided cognitive psychology from its very beginnings until the 1990s. On the one hand, at the dawn of computer science, inspired by the workings of computer operating systems and refusing to believe that human problem-solving could be understood simply as trial-and-error, the two young computer scientists Newell and Simon (1972) produced a version of cognitive psychology that thought of people as general problem-solvers whose problem-solving skills were independent of their content knowledge; it became known as information-processing psychology (IPP). On the other hand, inspired by the Swiss psychologist Jean Piaget and his Schema Theory (Piaget 2003), authors initially rallying under Jerome Bruner’s banner developed a branch of cognitive psychology concerned with the role of the activation of existing knowledge in knowledge acquisition; this became known as constructivist psychology (Hergenhahn 2001). The division of the Cognitive Psychology movement into these two irreconcilable halves paved the way for the disagreement between Barrows and Schmidt.

### Howard Barrows and the problem-solving skills approach to PBL

Although he may not have known it, Barrows owed the inspiration for his approach to PBL to the IPP school of thought. IPP was born in 1955, when Newell and Simon began their work in cognitive psychology from the premise that like computers, the human mind acts as a general problem-solving device (Newell et al. 1958). They believed that the process of solving a problem consisted in a collection of heuristic pathways that together formed a problem space and should be considered independently of the content of the problem. Newell and Simon’s research objective was to identify the invariant characteristics within the “Human Processing System” (Newell and Simon 1972).

While IPP was all the rage in the 1970s, by the 1980s it had hit an impasse. Firstly, from a methodological perspective, Newell and Simon’s trademark strategy for measuring the elusive “general problem-solving” capability was deeply flawed (Ohlsson 2012). It consisted in getting participants to voice their cognitive strategies out-loud while confronted with a sample problem. The experimenters recorded these verbal protocols, and then built computer programmes that mimicked the temporal order of the protocols in order to uncover the cognitive heuristics used by the human in this problem situation. However, there was a major problem with this approach: the verbal protocol was actually problem-specific rather than general—so much for their *General Problem Solver*. Secondly, from a theoretical perspective, the attempt to produce a general theory of problem solving didn’t work. Newell and Simon posited the existence of a problem-solving strategy that was context-free, but it became rapidly apparent that humans don’t generally engage in means-end analysis but use other cognitive strategies such as analogies, forward search etc., all of which are context-bound. And yet, perhaps because of psychology’s fascination with computers, the IPP model survived for decades longer than evidence should have allowed it to. Indeed, it survived long enough to spawn a model of medical problem-solving that ricocheted into the problem-based learning literature via Barrows: the hypothetico-deductive model.

The IPP methods were picked up by Arthur Elstein and Lee Shulman, working out of Michigan State University (Anderson 2003). In 1978, they attempted to demonstrate the existence of content-independent heuristics of medical problem-solving (Elstein et al. 1978). Clinicians, they conjectured, went through a process of hypothetico-deduction when faced with a medical problem. This meant that they would engage in the formulation of hypotheses for potential diagnoses, which would be either confirmed or disproved by new data from medical tests on the patient until the most likely hypothesis was left standing. The authors’ initial contention was that expert clinicians would fare better at hypothetico-deduction than novices, but their research found no evidence that expert clinicians were indeed better at generating hypotheses than novices. Instead, they were forced to acknowledge that the expert’s prior medical knowledge in the particular domain of the problem made a substantial difference in the expert clinicians’ ability to solve that problem, as compared with the novice. This indicated that the expertise was not one of deductive ability, but of content knowledge. However, the influence of IPP was such that they were not able to surrender the idea of the existence of content-independent heuristic processes. Instead of seeking a content-driven alternative explanation for the fact that some people appear to be better at problem-solving than others, they sought to explain this with the idea that some heuristics require extensive training.

The influence of the hypothetico-deductive model was then channelled into problem-based learning by Howard Barrows, particularly through his input into the McMaster curriculum in the 1970s and his 1980 book. Barrows began his research on hypothetico-deduction in the early 1970s, but his most developed argument in favour of content-independent reasoning processes can be found in *Problem*-*based Learning: An Approach to Medical Education*, the highly popular book on PBL which he co-authored with Robyn Tamblyn in 1980. In this book, the authors dismissed the idea that a physician’s clinical reasoning process was a mysterious intuitive “art”, and instead argued that these cognitive skills could and should be taught in medical education. The solution for this was to confront students with patient, health delivery, or research problems, since “by working with an unknown problem, the student is forced to develop problem-solving, diagnostic, or clinical reasoning skills” (p. 13).

Barrows argued that increased medical knowledge could even be detrimental to problem-solving skills as more precise knowledge might encourage students to tunnel-vision around what they had learned rather than consider a wider range of hypotheses. The distinction between content and process knowledge was cemented in Barrows’ call for process evaluations that are “concerned with the student’s ability to observe data, solve problems or show aspects of the clinical reasoning process, make clinical decisions and therapeutic decisions, and the like” (p. 113). Such aspects of the clinical reasoning process were made to include data perception and representation, problem formulation, hypothesis generation, inquiry strategy, diagnostic decisions, therapeutic decisions, time, cost, sequential management, and, finally, the medical information acquired. Therefore, while it would be unfair to claim that Barrows dismissed the importance of prior knowledge in problem-solving as Newell and Simon had, it is clear that the emphasis of his work was on the process of problem-solving via hypothesis generation. He believed that this process could be isolated enough from the specific problem content in which it was practiced to produce some general and teachable mechanisms by which medical problems should be approached; a trait which places Barrows squarely within the information-processing tradition.

This had some deep consequences for McMaster’s PBL curriculum. Beginning in 1977, calls were being issued by faculty and students to reform the 1969 curriculum (Roy 1978), and the process of change was taken over by Victor Neufeld, supported by Barrows. The new curriculum, progressively rolled out between 1977 and 1984, did away with the strong biomedical nature of the first curriculum and instead focused on priority healthcare problems management (MacDonald et al. 1989). Evidence of this change can be seen through the year-by-year evolution of the education materials found in the McMaster archives between 1975 and 1982, and in the notes of the Education Committee meetings (Ali 1977; Neufeld 1977) In the new curriculum, the students mainly dealt with long descriptions of patient cases compiled on the basis of lists of most commonly experienced medical issues, with a focus on solving the medical problem at hand. The objectives of the Faculty of Medicine were thus revised to read in top position: “to identify and define health problems at both an individual and a community level and to search for information to resolve or manage these problems” (Educational Programme Committee 1978). In addition, the development of clinical skills became a central feature of the reform efforts. Under the influence of Barrows and Tamblyn, the McMaster clinical skills training programme was constructed to train the students’ skills in encounters with simulated patients (Sutton 1977). This curriculum lasted until 1993, when, in the face of the high student failure rates in the national medical exam, McMaster abandoned the IPP approach and adopted a curriculum with many content-oriented features resembling those of Maastricht University (Norman et al. 2010).

### Henk Schmidt and the knowledge acquisition approach to PBL

The Knowledge Acquisition position owes a lot to the early works of Jean Piaget and Lev Vygotsky. Although Schmidt was most strongly influenced by the renaissance of constructivist ideas in the wake of the cognitive revolution, that renaissance would not have been possible without the groundwork laid out by Piaget’s Schema Theory (Piaget 1952, 1959, 2003). Piaget was the first to propose that knowledge is not stored as raw data but “constructed” through particular mental structuring processes called “schemas”. Schema Theory fell out of favour with the dominance of behaviourism in the 1960s, but by the late 1970s, a growing number of experimental psychologists, such as Andrew Ortony, Rand Spiro and David Ausubel, were looking into information encoding and retrieval in an attempt to explain the way knowledge is stored and reconstructed for recall. Even though they seldom explicitly referred to Piaget, they expanded on his notion of the schema by providing it with the scientific specificity that the Swiss psychologist was lacking. Under their pen, schemas were understood as mental “frames” or “scripts” that contained “slots” or “placeholders” that could be “instantiated” by elements in a situation (Anderson et al. 1978). Although all of these names made their way into Schmidt’s research on PBL in the late 1970s and early 1980s, the work of Richard Anderson returned with more consistency and force than the others. Schmidt recalled:

What (PBL) students were doing while discussing a problem was activating prior-knowledge in order to make sense of that problem. If the problem was sufficiently complex (but adapted to their level of knowledge) the need for new knowledge would arise and self-directed learning would satisfy that need. Since relevant prior knowledge was already activated, the new information would be more easily integrated. That this indeed leads to better learning is what I have shown in my PhD-thesis published in 1982 (personal communication).

In 1977, Anderson expanded on the concepts of “assimilation” and “accommodation” in Schema Theory (Anderson 1977). He posited that schemas could not be a simple aggregation of response components, perceptual features, semantic features, functional attributes and the like – instead, schemas could only be understood in terms of their emergent properties. This insight enabled Anderson to hypothesise how schemas are used (assimilation) and change (accommodation). He argued that accommodation happens as a gradual process whereby incongruent elements increasingly challenge an existing schema and make assimilation more and more difficult. Although people are extremely reluctant to accommodate their schemas, they also attempt to preserve cognitive consistency, and when the latter tendency wins over and a schema change is engaged, the acquisition of knowledge truthfully begins. Thus, Anderson saw accommodation as a *sine*-*qua*-*non* condition of learning:

I suspect that large-scale accommodation may be a dialectical process which entails a confrontation with difficulties in one’s current schema and coming to appreciate the power of an alternative (p. 429).

Anderson’s explanation paved the way for Schmidt’s idea that problems, by offering realistic situations for students to work with, could activate students’ existing schemas and thus provide the basis for sense-making that is essential to learning (Schmidt 1983a, b). The development of this theory was a slow process that began shortly after the opening of Maastricht Faculty of Medicine and ripened until 1983. We can see from archival evidence that the research efforts began in earnest in 1977, although at the time the education research group’s ideas on learning in PBL were a little haphazard. A note in the tutor training manual *Het Tutorensysteem* indicates that the researchers believed that the strength of PBL lay in the promotion of knowledge retention and transfer, but without further specification (Bouhuijs et al. 1977). In fact, the text indicates that the authors, including Schmidt and his colleague Peter Bouhuijs, were aware of the limitations of contemporary research in the field. By 1979, Schmidt had developed more precise ideas on this. He elaborated on his previous work with a paper entitled *Leren met Problemen* (1979) and for the first time referred to the activation of prior knowledge and Ausubel’s take on Schema Theory. At this point, Schmidt’s work was fully aligned with the constructivist credo that people do not passively ingest the outside world but instead constantly attempt to give meaning to it through personal interpretations of what their senses tell them. In a paper from 1983, he offered three connected explanations of the learning process that takes place in PBL: the activation of prior knowledge; encoding specificity (the similarity between the situation in which knowledge is learned and the situation is which it is applied); and elaboration of knowledge (Schmidt 1983a, b). By this stage, his research had expanded well beyond the work of Anderson and Ausubel and was aggregating reports from all over the blooming field of cognitive psychology. Schmidt’s later article on the foundations of problem-based learning provided some elaborations of these three ideas, but the central themes remain the same to this day (Schmidt 1993).

### How the dispute played out at Maastricht University

**Fig. 1:**
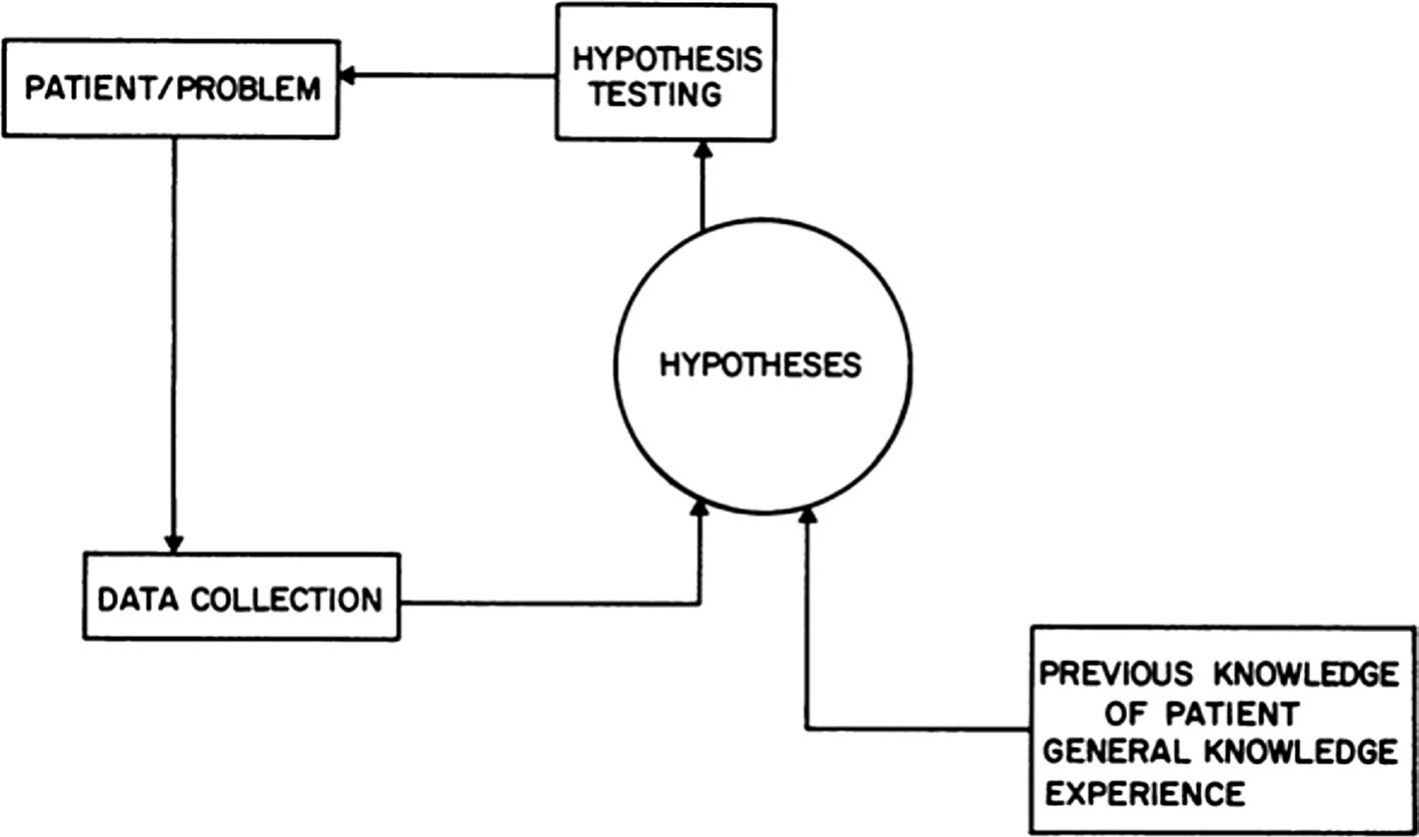
The hypothetico-deductive model of Feightner et al. (1977)

The story takes root in the early 1970s, when Howard Barrows took it upon himself to demonstrate that educational aids could be used to improve “clinical reasoning skills”, “problem-solving skills”, “diagnostic skills” and other variations thereof. The first apparent results of this research appeared in 1972, under the title *The diagnostic (problem*-*solving) skill of the neurologist*, in which it was claimed that hypothetico-deduction could be equated to a “cognitive hat rack” for organising the information acquired during the patient interview (Barrows and Bennett 1972). Barrows worked closely with Vic Neufeld on this research—neither of them having a prominent role in the curriculum development at McMaster at that time. Neufeld studied medical education at Michigan State University, where Elstein and Shulman were doing their work and according to their research assistant Geoffrey Norman, the Barrows-Neufeld duo “had a close relation” with the Elstein–Shulman team (personal communication). It is therefore unsurprising that Barrows borrowed so heavily from the theory of hypothetico-deduction to support his ideas. This research culminated in a paper written in 1977, in which not only was the “hat rack” idea alive and well, but prior knowledge was relegated to a secondary relevance (Feightner et al. 1977). They developed a model of medical problem solving which would be of crucial importance in the later debates on PBL (Fig. 1).

The McMaster team boldly concluded: “Family physicians do have identifiable legitimate problem solving skills which they can teach. We feel that the model outlined above can help student to develop their clinical problem solving skills” (p. 71). These are the ideas with which Barrows and Neufeld travelled to Maastricht to act as educational consultants to the new Faculty of Medicine. There is ample evidence from written correspondence between them that throughout the 1970s and 1980s, Schmidt held both Barrows and Neufeld in very high esteem (Schmidt 1983a, b). In Schmidt’s eyes, Barrows was one of the founders of PBL and therefore warranted listening to. It is therefore not surprising to find Barrows and Neufeld’s model of hypothetico-deduction in Schmidt’s early work. How did Schmidt move from one paradigm to the other? A comparison of his two major contributions between 1977 and 1979 may provide answers to this question. In 1977, Schmidt wrote *Probleemgeoriënteerd onderwijs*, a booklet designed to be used internally at the Faculty (Schmidt 1977a, b). In it, he wrote down for the first time his ideas on the cognitive mechanisms underlying learning through PBL. This manuscript was published 1 year later in the Dutch journal *Metamedica* (Schmidt 1978), and 1 year later re-written in a substantially amended format as *Leren met Problemen* (Schmidt 1979). The key lies in the changes made between the 1977 paper (and its identical reprint in 1978) and the 1979 paper. The table below indicates the most significant of these changes. It may seem strange that Schmidt offered argument from both paradigms in his work, even though they are not epistemologically compatible—but this incompatibility was not generally understood at the time, even among cognitive scientists (Table 1).

**Table 1:** Information processing versus constructivist content in Schmidt (1977a, 1979)

Title, date of paper	These are the observations that Schmidt made pertaining to the information processing paradigm	These are the observations that Schmidt made pertaining to the constructivist paradigm
*Probleemgeoriënteerd onderwijs* (1977)	(1) Schmidt made reference to computer-simulations as latest advances in cognitive sciences (2) Schmidt used Barrows and Tamblyn (1976) model of clinical reasoning with hypothesis generation (3) Schmidt cited a belief that problem-solving is the “transformation of a problem into a solution by *hypothesis*-*testing*.” (4) Schmidt argued that problem-solving can mean the *pathway* through which a solution can be brought forward even if the underlying mechanisms are not fully known (5) Schmidt claimed that the central advantage of PBL is the training of *problem*-*analysis skills* and promotion of thinking processes IPP References in Schmidt’s bibliography: Barrows and Mitchell (1975), Barrows and Tamblyn (1976), Feightner et al. (1977), Elstein et al. (1972), Gorry (1970)	(a) Schmidt interpreted Elstein (1972) as indicating importance of prior experience for medical problem solving (b) Schmidt recognized the work of De Groot (though he did not reference it) in demonstrating that good chess players make poor doctors due to the importance of *prior knowledge* (c) Schmidt proposed that PBL is about understanding the *underlying mechanisms* of phenomena Constructivist references in Schmidt’s bibliography: Bruner et al. (1956)
*Leren met problemen* (1979)	(6) Schmidt argued that students and doctors generate hypotheses automatically, in the same way. This is not a skills that can trained or improved independently of content. IPP References in Schmidt’s bibliography: Barrows (1968)	(d) Schmidt argued that the *Activation* of prior knowledge through self-study allows students to “fill up” relevant cognitive structures (e) Schmidt stated that internal representation of knowledge is a construction and interpretation of reality (f) He further stated that these constructions are tested against reality, people make theories based on knowledge, test them, then make other theories Constructivist references in Schmidt’s bibliography: Ausubel (1968), Bruner (1971), Bruner et al. (1966), de Groot (1965), Kelly (1955), Miller (1960)

We see in the 1977 paper an extensive explanation of PBL in terms of Barrows, Elstein and Shulman’s hypothetico-deductive model, with diagrams that closely resemble those published by Barrows in his own work from 1977. And yet, already in 1977, Schmidt was intrigued by the experiments of a Dutch psychologist, De Groot (1965), on chess players. De Groot had tested chess players’ ability to solve a checkers problem, and found that chess masters made mediocre checkers players—indicating the absence of a general problem-solving ability among chess masters. The plausible explanation was that chess masters had a great prior knowledge of possible chess combinations to draw from when solving chess problems, that was of no use to them when solving a checkers problem. Schmidt concluded, as Elstein also did later, that prior knowledge must be a major factor in performance on problem-solving tasks. But these ideas could only be considered hunches at the time: aside from a passing mention of Bruner, in 1977, Schmidt’s reference list is remarkably devoid of constructivist literature. This was very much amended in the 1979 paper, which is replete with notes on Ausubel, Kelly, De Groot, as well as digressions on Bruner and Miller. Although Schmidt had used the term “prior knowledge” before, this was the first time that he framed it strongly in terms of the “activation of prior knowledge”—and therefore PBL as a learning method that could be used precisely for that purpose. By 1979, gone were the references to hypothetico-deduction, absent the diagrams of Barrows–Schmidt now saw hypothesis generation as an automatic process of the human cognitive architecture which therefore cannot not be trained, and he therefore saw little point in expending energy researching it.

### Incompatible approaches to PBL

Barrows and Schmidt were in regular contact during the 1980s as consultants from McMaster flew to Maastricht and vice versa. In particular, in 1983, Schmidt organised a symposium on PBL for which he invited Barrows as a speaker. A series of letter exchanges leading up to this event sheds some light on their academic relationship. For instance, a letter written by Barrows to Schmidt in July 1982 indicates that the former believed PBL to be the acquisition of basic sciences knowledge and “medical problem solving as a cognitive skill” in equal measure (Barrows 1982). In response, Schmidt returned a letter to Barrows in January 1983, in which he voiced in the clearest way possible the rift between their approaches to PBL:

I think that the difference between your work and mine is more a difference of problem-solving in terms of encoding, storage and retrieval of knowledge for use in problem-solving situations (and, most important, in terms of the organization of knowledge in memory), while you focus on the process of problem-solving itself. My main interest lies in the role PBL plays in knowledge acquisition - that is why I refer with emphasis to theories of learning (role of knowledge, inference production, organization of knowledge, retrieval cues etc.) - while you are particularly interested in how the students use the knowledge acquired in clinical problem-solving situations (and therefore refer to theory and research in that area). In fact, I think that our approaches are complementary. We would make a good team! When you are in Maastricht, we certainly should sit down to discuss these matters and others (Schmidt 1983a, b).

In fact, it seems that the approaches were not so much complimentary as mutually exclusive as they relied on opposing understandings of the role of knowledge in problem-solving. The version of PBL supported by Barrows posited the primacy of heuristics and associations in medical problem-solving. That of Schmidt relied on problem-solving anchored in prior knowledge and experience. But problem-solving cannot be both content-independent and content-dependent—these two positions are therefore epistemologically incompatible with one another. Therefore either Barrows or Schmidt was right about PBL, but they could not both be. This incompatibility is not a matter of a historical clash of personalities: by all accounts, Schmidt and Barrows actually held each other in high regard. This is really a question of two interpretations of PBL, the underlying epistemological constructs of which are irreconcilable, and produced a very different type of problem in PBL. Whereas a PBL problem for Barrows could be “a written case, case vignette, standardized (also called simulated patient), computer simulation, video tape” (Barrows and Tamblyn 1980, p. 5), for Schmidt, a problem could also look like a description of a biomedical phenomenon with no “solution”. Schmidt’s problems required instead that the underlying phenomena or theory be understood by the students (Schmidt 1993). While this sort of problem could be and was translated to almost any academic discipline, problems based on the management of healthcare problems could not be. Perhaps this serves to explain the profusion of PBL programmes in the Netherlands in all manner of academic disciplines ranging from psychology to liberal arts.

Luckily, History has provided us with some answers as to which of the two versions of PBL fared the best in terms of helping students to solve medical problems; by the mid-1980s, IPP was beginning to crumble as a psychological paradigm. In 1985, Christine McGuire lamented resilience of the idea of content-independent cognitive skills and abilities:

Professional evaluators (…) wanted to believe in the existence of some generalized kind of cognitive achievement – a related set of skills or developed abilities – that individuals could bring to bear in managing patient problems. They have been pursuing that chimera ever since, despite a mind-numbing torrent of studies that continue monotonously to report the same findings (McGuire, 1985).

McGuire also stated that she did not believe that the doctors reported in Barrows’ studies were actually engaging in hypothetico-deduction:

Doubts that these diagnostic labels are genuine hypotheses are considerably exacerbated if, as Barrows and Tamblyn say, they literally ‘pop’ into the clinician’s head within moments of the initial encounter. Such a process appears to be more akin to the act of pattern-matching or to the procedure involved in comparing group phenomena with various templates and selecting best fit (McGuire, 1985).

In what should have been a death blow to the Problem-Solving Skills approach to PBL, in 2002, Elstein became his own harshest critic when he acknowledged that the theory that medical problem-solving was based on hypothetico-deduction processes was in large part erroneous (Elstein and Schwarz 2002). Most problems, he realised, were actually resolved on the basis of pattern recognition or the construction of a mental model of the problem. Both of these processes were based on the extent of the clinician’s knowledge rather than the mastery of heuristics. This, he acknowledged, has such strong implications for problem-based learning that it led him to a re-evaluation of the purpose of the method:

The finding of case specificity showed the limits of teaching a general problem solving strategy. Expertise in problem solving can be separated from content analytically, but not in practice. This realisation shifted the emphasis towards helping students acquire a functional organisation of content with clinically usable schemas. This goal became the new rationale for problem based learning (p. 731).

This is not quite a full embrace of the constructivist paradigm, as Elstein struggled to let go of his embrace of IPP “analytically”. Yet ironically, “in practice”, Elstein turned to the alternative approach to PBL: constructivism and the Knowledge Acquisition approach championed by Henk Schmidt. And yet, despite these strong criticisms including from within the school of thought of hypothetico-deduction, Barrows refused to re-evaluate his approach to PBL. In the light of this, the divergence with Schmidt that had begun in the late 70s turned into a dispute in the late 1980s, culminating in an open confrontation during a review of the PBL curriculum of Sherbrooke University in Canada in 1992, as Schmidt recalls:

Howard Barrows, George Bordage, Charles Boelen (of the World Health Organization), and I were invited around 1992 to assess the then five-year old problem-based medical curriculum of the University of Sherbrooke in Canada. I had been one of this school’s consultants, had visited many times in the previous years, and had conducted teacher training workshops emphasizing PBL as a method to acquire knowledge and its embedding in cognitive constructivism. Barrows (perhaps not aware of my previous role) was highly critical about what had been accomplished, because the curriculum “was not a problem-solving curriculum”. Much more emphasis should be put on students acquiring the process of clinical reasoning, otherwise it was not really problem-based. I felt it necessary to object and eventually ended up in a heated argument with him (personal communication).

When asked, Georges Bordage and Charles Boelen could not remember the specifics of this particular event, but both agreed that Barrows, on different occasions, “was not too enthusiastic about knowledge-based PBL- too much about knowledge and not enough about the process of clinical reasoning, same issue” (Bordage, personal communication). Boelen recalled:

On another occasion at UNM in Albuquerque, I think in 1993, as we were considering with a dozen of colleagues PBL applied to public health problems, I remember him exposing very strongly the same arguments and the conversation became so heated that our friend Charles Engel [a PBL pioneer in Australia] who dared to argue was shocked and about to weep (personal communication).

If any doubt persists within the reader, an analysis of the later works of Barrows clearly show that he espoused information-processing to some degree until the end of his academic career, a claim also confirmed by his former research assistant Norman (personal communication). In 1996, Barrows produced a paper summarising his view of PBL in which he re-iterated the importance of clinical problem-solving skills, but also the importance of the acquisition of a medical knowledge-base that would be integrated, centred around the cues of patient problems, and enmeshed with the problem-solving process (Barrows 1996a, b).

## Conclusion

Until the end, Barrows and Schmidt retained two different understandings of the role of problems in PBL. Even though this debate was settled at Maastricht, it remains a major source of debate in PBL education and research around the world. As reported by Schmidt and colleagues, there are still many PBL curricula that follow the information-processing approach to PBL (Schmidt et al. 2009). In these curricula, PBL is still seen as a method for developing problem-solving skills rather than as a vehicle for understanding the underlying principles or mechanisms that produce these phenomena. These two interpretations are at such odds with one another that calling both of them “problem-based learning” tends to deprive PBL of its psychological and philosophical underpinnings and may leave only a methodological shell behind, devoid of theoretical support. From a historical perspective, declaring a victor in the dispute for the interpretation of PBL is a difficult matter. In terms of scientific consensus, the constructivist Maastricht interpretation of PBL, as the more theoretically-grounded approach can be awarded a clear academic victory: information-processing has largely been erased from the psychology of learning. But in terms of educational practice, educators from all over the world continue to preach the teaching of general problem-solving skills, so the turf-war for the interpretation of PBL is far from over.

Whilst as a historian, one would not purport to prescribe the application of historical lessons to the present day, one may very well encourage present day educators to seriously and extensively question the rationale behind their PBL curriculum: is it primarily grounded in information-processing or in constructivism, and is there a full and open understanding of the educational consequences that this implies?

This research was limited in scope to McMaster and Maastricht in the field of medical education, but there may be other disciplines and areas where the debate between IPP and constructivism played out. This may be worthwhile investigating. This being a historical paper, it has not investigated the present situation with regards to Problem-solving and Knowledge Acquisition curricula, including at McMaster and Maastricht; this may be an interesting exercise for follow-up research.

## References

[CR1] Ali M (1977). To: M.D. Education Committee—January 7, 1977. Educational Programme Committee—1977/1978, Box 233.2;1.

[CR2] Anderson R, Anderson R, Spiro R, Montague W (1977). The notion of schemata and the educational enterprise: General discussion of the conference. Schooling and the acquisition of knowledge.

[CR3] Anderson WA (2003). Arthur S. Elstein, Ph. D.: Skeptic, scholar, teacher and mentor. Advances in Health Sciences Education.

[CR4] Anderson RC, Spiro RJ, Anderson MC (1978). Schemata as scaffolding for the representation of information in connected discourse. American Educational Research Journal.

[CR5] Ausubel DP (1968). Education psychology: A cognitive view.

[CR70] Barrows HS (1968). Simulated patients in medical teaching. Canadian Medical Association Journal.

[CR6] Barrows HS (1982). Letter to Henk Schmidt.

[CR7] Barrows HS (1996). In memoriam: James E. Anderson, MD. Teaching and Learning in Medicine.

[CR8] Barrows HS (1996). Problem-based learning in medicine and beyond: A brief overview. Teaching and Learning.

[CR9] Barrows HS, Bennett K (1972). The diagnostic (problem solving) skill of the neurologist: Experimental studies and their implications for neurological training. Archives of Neurology.

[CR10] Barrows HS, Mitchell DLM (1975). An innovative course in undergraduate neurosciences, experiment in problem-based learning with ‘problem-boxes’. British Journal of Medical Education.

[CR11] Barrows HS, Tamblyn RM (1980). Problem-based learning: An approach to medical education.

[CR12] Barrows, H. S., & Tamblyn, R. M. (1976). Guide to the development of skills in problem-based learning and clinical (diagnostic) reasoning. Monograph #1. McMaster University Faculty of Health Sciences.

[CR13] Bartholomeus P (1977). Het Skillslab in de Komende Jaren [the Skillslab in coming years]. OC 77-069; 07.C06 - inventaris 94..

[CR14] Bechtel W, Abrahamsen A, Graham G, Smelser NJ, Baltes PB (2001). Cognitive science, history. International encyclopedia of the social and behavioral sciences.

[CR15] Bouhuijs P, Bremer J, Metsemakers J, Schmidt H, Vusse GV (1977). Het tutorensysteem [The Tutor System].

[CR71] Bruner JS (1971). “The Process of Education” Revisited. The Phi Delta Kappan.

[CR16] Bruner JS, Goodnow JJ, Austin GA (1956). A study of thinking.

[CR17] Bruner JS, Oliver RR, Greefield PM (1966). Studies in cognitive growth.

[CR18] Campbell EJ (1973). The McMaster Medical School at Hamilton. Ontario. The Lancet.

[CR19] Chomsky N (1956). Three models for the description of language. IRE Transactions on Information Theory.

[CR20] Chomsky N, Jakobovits LA, Miron MS (1967). A review of B. F. Skinner’s verbal behavior. Readings in the psychology of language.

[CR21] de Groot AD (1965). Thought and choice in chess.

[CR22] Educational Programme Committee. (1978). Objectives of the M.D. Programme (Revised). *Educational Programme Committee*—*1977/1978, Box 233.2;4*. Hamilton, ON: McMaster University FHS/HHS Archives.

[CR23] Elstein AS, Kagan N, Schulman LS, Jason H, Loype MJ (1972). Methods and theory in the study of medical inquiry. Journal of Medical Education.

[CR24] Elstein AS, Schwarz A (2002). Clinical problem solving and diagnostic decision making: Selective review of the cognitive literature. British Medical Journal.

[CR25] Elstein A, Shulman L, Sprafka S (1978). Medical problem solving.

[CR26] Evans, J. (1966). General objectives. In *Objectives of the Faculty School of Medicine, Box 145.8;1*. Hamilton, ON: McMaster University HHS/FHS Archives.

[CR27] Feightner JW, Barrows H, Neufeld V, Norman G (1977). Solving problems: How does the family physician do it?. Canadian Family Physician.

[CR28] Garvin D (2003). Making the case. Harvard Magazine.

[CR29] Gorry GA (1970). Modelling the diagnostic process. Journal of Medical Education.

[CR30] Hergenhahn BR (2001). An introduction to the history of psychology.

[CR31] Kelly GA (1955). The psychology of personal constructs.

[CR32] Knowles MS (1975). Self-directed learning: A guide for learners and teachers.

[CR33] MacDonald PJ, Chong PJ, Chongtrakul P, Neufeld VR, Tugwell P, Chambers LW, Oates MJ (1989). Setting educational priorities for learning the concepts of population health. Medical Education.

[CR34] Mager RF (1962). Preparing instructional objectives.

[CR35] McAuley, J. (1979). McMaster oral history—Dr. J.R. Evans—28th September 1979. In *Founding fathers interviews*. Hamilton, ON: McMaster University FHS/HHS Archives.

[CR36] McGuire C (1985). Medical Problem-Solving: A critique of the literature. Journal of Medical Education.

[CR37] Miller GA (2003). The cognitive revolution: A historical perspective. Trends in Cognitive Sciences.

[CR38] Miller GA, Galanter E, Pribam KH (1960). Plans and the structure of behaviour.

[CR39] Moust J, Schmidt H, Bouhuijs P (2007). Introduction to problem-based learning: A guide for students.

[CR40] Neufeld, V. R. (1977). Ref: Proposed terms of reference for MD Program task force on objectives—To: Ron McAuley—Feburary 8, 1977. *Letter from 1977. Educational Programme Committee*—*1977/1978, Box 233.2;1*. Hamilton, ON: McMaster University FHS/HHS Archives.

[CR41] Neufeld VR, Barrows HS (1974). The “McMaster philosophy”: An approach to medical education. Journal of Medical Education.

[CR42] Neufeld VR, Spaulding WB (1973). Use of learning resources at McMaster University. The British Medical Journal.

[CR43] Newell A, Simon HA (1972). Human problem solving.

[CR44] Newell A, Simon HA, Shaw JC (1958). Elements of a theory of human problem solving. Psychological Review.

[CR45] Norman GR, Neville AJ, Blake JM, Mueller CB (2010). Assessment steers learning down the right road: Impact of progress testing on licensing examination performance. Medical Teacher.

[CR46] Ohlsson S (2012). The problems with problem solving: Reflections on the rise, current status, and possible future of a cognitive research paradigm. The Journal of Problem Solving.

[CR47] Piaget J (1952). The origins of intelligence in children.

[CR48] Piaget J (1959). The language and thought of the child.

[CR49] Piaget J (2003). The psychology of intelligence.

[CR50] Limburg Rijksuniversiteit (1972). Basisfilosofie Achtste Medische Faculteit. Medisch Contact.

[CR51] Roy, W. (1978). Exit survey report—Class of 1977. *Educational Programme Committee*—*1977/1978, box 233.2;4*. Hamilton, ON: McMaster University FHS/HHS Archives.

[CR52] Schmidt HG (1977). Probleemgeoriënteerd onderwijs.

[CR53] Schmidt, H. G. (1977b). Tutortraining—De Taken van de Tutor [Tutor training, the tasks of the tutor]. *OC 77*-*164; 07*-*C06*—*inventaris 98*. Maastricht: Rijksarchief in Limburg.

[CR54] Schmidt HG (1978). Probleem-georiënteerd onderwijs: leren aan de hand van problemen. Metamedica.

[CR55] Schmidt, H. G. (1979). Leren met problemen: een inleiding in probleemgestuurd onderwijs. *Working Paper*. Maastricht: Maastricht University.

[CR56] Schmidt H (1983). Letter to H.S. Barrows—7 January 1983.

[CR57] Schmidt HG (1983). Problem-based learning: Rationale and description. Medical Education.

[CR58] Schmidt HG (1993). Foundations of problem-based learning: Some explanatory notes. Medical Education.

[CR59] Schmidt H, Bouhuijs P (1980). Onderwijs in taakgerichte groepen [Education in task-oriented groups].

[CR60] Schmidt H, Majoor G, Wijnen W (1979). Introduction to the medical study.

[CR61] Schmidt HG, Van de Molen HT, Te Winkel WW, Wijnen WH (2009). Constructivist, problem-based learning does work: A meta-analysis of curricular comparisons involving a single medical school. Educational Psychologist.

[CR62] Servant, V. F. (2016). Revolutions and Re-iterations: An intellectual history of problem-based learning. *Unpublished doctoral thesis*. Rotterdam: Erasmus University Rotterdam.

[CR63] Servant-Miklos VFC, Spliid CM (2017). The construction of teaching roles at Aalborg university centre, 1970–1980. History of Education.

[CR64] Spaulding, W. B. (1968). The undergraduate medical curriculum: McMaster University. *Objectives of the Faculty School of Medicine*—*Box 145.8;1*. Hamilton, ON: HHS/FHS Archives.

[CR65] Spaulding WB (1969). The undergraduate medical curriculum: McMaster University. Canadian Medical Association Journal.

[CR66] Spaulding WB (1991). Revitalizing medical education, McMaster medical school the early years 1965–1974.

[CR67] Spaulding WB, Neufeld VR (1973). Regionalization of medical education at McMaster University. The British Medical Journal.

[CR68] Sutton, J. (1977). To: Dr. G.S. Cameron—November 15, 1977. *Educational Programme Committee*—*1977/1978, Box 233.2;3*. Hamilton, ON: McMaster University FHS/HHS Archives.

[CR69] Williams G (1980). Western reserve’s experiment in medical education and its outcomes.

